# Cytotoxic effects of caffeic acid undecyl ester are involved in the inhibition of telomerase activity in NALM-6 human B-cell leukemia cells

**DOI:** 10.3892/ol.2013.1482

**Published:** 2013-07-23

**Authors:** AYAKO TOMIZAWA, SYU-ICHI KANNO, YUU OSANAI, SHIN YOMOGIDA, MASAAKI ISHIKAWA

**Affiliations:** Department of Clinical Pharmacotherapeutics, Tohoku Pharmaceutical University, Sendai, Miyagi 981-8558, Japan

**Keywords:** caffeic acid, telomerase reverse transcriptase, cytotoxicity, telomerase, NALM-6

## Abstract

Our previous study reported that caffeic acid undecyl ester (CAUE) has a potent cytotoxic effect and induces apoptosis in NALM-6 cells, but not in normal human lymphocytes. The majority of normal human cells have no detectable telomerase activity, however, activity is commonly detected in cancer cells. Thus, inhibiting telomerase activity and inducing apoptosis may have a selective effect on cancer cells. The aim of the present study was to investigate the inhibitory effects of telomerase activity by CAUE in a NALM-6 cell culture system. CAUE was shown to preferentially damage DNA synthesis compared with RNA or protein synthesis. In addition, telomerase activity was significantly suppressed and the activity of human telomerase reverse transcriptase (hTERT), a subunit of telomerase, was decreased following treatment with CAUE, each in a concentration-dependent manner. These results indicated that the cytotoxic effects of CAUE are mediated by the inhibition of DNA synthesis and telomerase activity. The present study is the first to identify the cytotoxic mechanisms of CAUE in leukemia cells.

## Introduction

Telomerase, a specialized ribonucleoprotein, plays an essential role in cell proliferation by protecting against the problem of end-replication by adding TTAGGG repeats to telomeres (1). The majority of normal human cells have no detectable telomerase activity, however, activity is commonly detected in cancer cells (2,3). The inhibition of telomerase causes a progressive and critical reduction of telomeres, leading to a potent signal for the blockage of cell proliferation and the induction of apoptosis (4). Targeting the inhibition of telomerase activity and the induction of apoptosis may have a selective effect on cancer cells. Clinically, B-cell acute lymphoblastic leukemia is curable, however, ≥50% of adults experience treatment failure as a consequence of drug resistance and the inability of older adults to tolerate the side-effects of therapy (5). Therefore, it is desirable to develop novel anticancer drugs against B-cell leukemia, including those targeting the inhibition of telomerase activity, to prevent side-effects following chemotherapy. Our previous study reported that treatment with caffeic acid undecyl ester (CAUE), a novel caffeic acid derivative, reduced cell survival in human B-cell leukemia NALM-6 cells, but exhibited no significant effect on the survival of normal lymphocytes. In addition, the cytotoxic induction mechanisms of CAUE were shown to be involved in the intrinsic apoptotic pathway in a caspase-dependent manner (6). The present study focused on the inhibitory effects of telomerase activity by CAUE in a NALM-6 cell culture system.

## Materials and methods

### Materials and cell culture

CAUE was prepared as described previously (7). All other reagents, unless otherwise stated, were of the highest grade available and purchased from Sigma-Aldrich (St. Louis, MO, USA) or Wako Pure Chemical Industries, Ltd. (Osaka, Japan). Antibodies against human telomerase reverse transcriptase (hTERT; rabbit polyclonal; Santa Cruz Biotechnology, Inc., Santa Cruz, CA USA) and β-actin as the loading control (rabbit polyclonal; Cell Signaling Technology, Inc., Danvers, MA, USA) were used. Human B-cell leukemia NALM-6 cells were supplied by the Cell Resource Center for Biomedical Research (Tohoku University, Sendai, Japan). Cell culture reagents were obtained from Invitrogen Life Technologies (Carlsbad, CA, USA) and the cells were routinely cultured using standard methods, as described previously (8,9).

### DNA, RNA and protein synthesis assays

The effect of CAUE on the synthesis of DNA, RNA and protein was determined by incorporation of the radioactive precursors [^3^H]-thymidine, [^3^H]-uridine and [^14^C]-leucine (GE Healthcare, Amersham, UK). Briefly, 4×10^5^ cells/ml were cultured in 96-well round-bottom plates in a total volume of 100 μl culture medium with or without the indicated concentrations of CAUE. Following incubation for 4 h, [^3^H]-thymidine (37 MBq/ml), [^3^H]-uridine (37 MBq/ml) or [^14^C]-leucine (1.85 MBq/ml) were added, each corresponding to a total activity of 148 Bq, and incubated for an additional 90 min. The cells were harvested on filter membranes using a Labo Mash cell harvester (Futaba Medical Inc., Tokyo, Japan). Subsequent to drying, the radioactivity of the material was measured by a LS-6500 liquid scintillation β-counter (Beckman Coulter, Miami, FL, USA).

### Telomerase activity assay

Telomerase activity was measured using a stretch PCR-based TeloChaser system (Toyobo Co., Ltd., Osaka, Japan), according to the manufacturer’s instructions. Briefly, 4×10^5^ cells were lysed in 50 μl lysis reagent and incubated on ice for 20 min. Following centrifugation at 12,000 × g for 20 min, DNA products were isolated and 26 cycles of PCR amplification were performed at 95°C for 30 sec, 68°C for 30 sec and 72°C for 45 sec. PCR products were electrophoresed on a 10% polyacrylamide gel and stained with ethidium bromide. Images were captured using the FLA-3000G image analyzer (Fujifilm Corp., Tokyo, Japan).

### Western blotting

The effects of cellular signal transduction on hTERT protein expression by CAUE were determined by western blotting (10). Briefly, the cells were incubated with the indicated concentrations of CAUE, washed with phosphate-buffered saline (PBS) and lysed. Protein concentrations were measured using the BCA™ protein assay kit (Thermo Fisher Scientific Inc., Rockford, IL, USA), according to the manufacturer’s instructions. Samples of each protein (30 μg) were loaded onto 7.5% sodium dodecyl sulfate-polyacrylamide gels. Following electrophoresis, the protein was transferred to polyvinylidene difluoride membranes and blocked with Blocking One^®^ (Nacalai Tesque, Inc., Kyoto, Japan) for 1 h, prior to incubation with antibody overnight at 4°C. The membranes were then washed with wash buffer (PBS containing 0.05% Tween 20) and incubated with horseradish peroxidase-linked secondary antibody for 1 h. Subsequent to being washed with wash buffer, the protein levels were analyzed by enhanced chemiluminescence using Pierce^®^ western blotting substrate (Thermo Fisher Scientific Inc.).

### Statistical analysis

Statistical analysis was performed using a one-way analysis of variance, followed by Williams’ multiple comparison test. P<0.01 was considered to indicate a statistically significant difference.

## Results

### Effects of CAUE on DNA, RNA and protein synthesis

**Figure 1:**
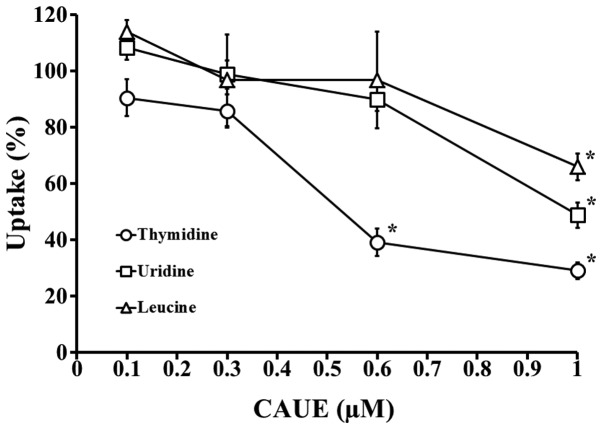
Effect of CAUE on DNA, RNA and protein synthesis in NALM-6 cells. Cells (4×10^4^) were cultured in 96-well round-bottom plates with the indicated concentrations of CAUE for 4 h in a total volume of 100 μl. ^3^H-thymidine, ^3^H-uridine and ^14^C-leucine were applied. Incorporation of labeled substrates are presented as percentage vs. control (CAUE vehicle). CAUE initially demonstrated inhibitory effects on DNA replication at 0.6 μM; however, there was no significant effect on RNA and protein synthesis at this concentration. Results are presented as mean ± SEM. ^*^P<0.01 vs. control group. CAUE, caffeic acid undecyl ester.

To investigate the cytotoxic mechanisms of CAUE, the kinetics of macromolecule synthesis were examined (Fig. 1) and the incorporation of radiolabeled substrates into DNA, RNA and protein was monitored. No effect was identified on CAUE at concentrations of <0.3 μM, however, CAUE showed significant inhibition of DNA replication at 0.6 μM (39.1% vs. CAUE vehicle group). In addition, no effects were identified on RNA and protein synthesis. Following treatment with higher concentrations of CAUE (1 μM), the DNA, RNA and protein levels significantly decreased to 29.0, 48.8 and 65.9%, respectively.

### Effects of CAUE on telomerase activity and expression of hTERT

**Figure 2:**
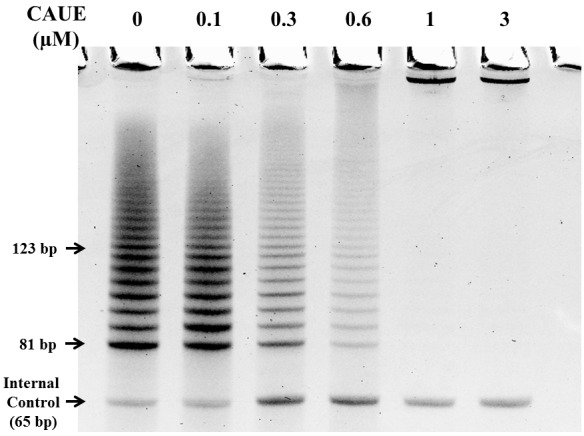
Effect of CAUE on telomerase activity. Cells were incubated with the indicated concentrations of CAUE for 18 h and telomerase activity was measured by stretch PCR. Percentage inhibition of telomerase was calculated from the band intensity and the results revealed that telomerase activity, compared with the CAUE vehicle group (100%) decreased to 92, 64, 19 and 0% following treatment with 0.1, 0.3, 0.6 and 1 μM CAUE, respectively. Similar results were obtained in three separate sets of experiments. CAUE, caffeic acid undecyl ester.

**Figure 3:**
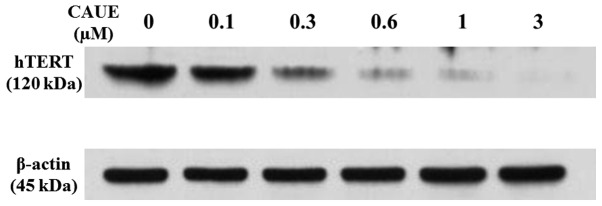
Effect of CAUE on hTERT protein expression. Cells were incubated with the indicated concentrations of CAUE for 18 h and hTERT protein expression was determined by western blotting. At 0.1, 0.3, 0.6 and 1 μM CAUE, hTERT expression was 96, 48, 11 and 7%, respectively, as determined by densitometry analysis (compared with the CAUE vehicle group as 100%). Similar results were obtained in three separate sets of experiments. CAUE, caffeic acid undecyl ester; hTERT, human telomerase reverse transcriptase.

To examine the effects of CAUE on telomerase activity, the NALM-6 cells were incubated in the absence (CAUE vehicle) or presence of CAUE. Telomerase activity was measured by stretch PCR (Fig. 2) and expressed as a ladder of 6-bp bands or multiples of 6-bp intervals. Telomerase activity was significantly suppressed following treatment with CAUE in a concentration-dependent manner when compared with the untreated cells. The percentage inhibition of telomerase was calculated using the band intensity, and the results revealed that when compared with that of the CAUE vehicle group (100%) telomerase activity decreased to 92, 64, 19 and 0% following treatment with 0.1, 0.3, 0.6 and 1 μM CAUE, respectively. To verify the mechanisms for the inhibitory effect of CAUE on telomerase activity, the telomerase-component gene was investigated in the NALM-6 cells to determine if CAUE was able to modulate its expression. The hTERT subunit of telomerase functions as a critical determinant of enzyme activity (11), therefore, changes in hTERT protein expression due to CAUE treatment were examined by western blotting. As presented in Fig. 3, CAUE induced a concentration-dependent decrease in hTERT expression compared with the CAUE vehicle group (100%). At concentrations of 0.1, 0.3, 0.6 and 1 μM CAUE, hTERT expression was 96, 48, 11 and 7%, respectively, as determined by densitometry analysis. The highest concentration of CAUE (3 μM) showed complete inhibition of telomerase activity (Fig. 2) and hTERT expression (Fig. 3).

## Discussion

Our previous study demonstrated that CAUE exhibited potent cytotoxic effects on human B-cell leukemia NALM-6 cells, but not on normal human lymphocytes (6). Activated B cells exhibit significantly longer telomeres and increased telomerase activity (12). The present study aimed to investigate the cytotoxic mechanisms of CAUE in NALM-6 cells and, as shown in Fig. 1, CAUE exhibited preferential damage to DNA synthesis compared with RNA and protein synthesis. This indicated that CAUE directly affects the nucleus and impairs DNA synthesis, resulting in the induction of apoptosis.

Caffeic acid phenethyl ester is a parent compound of CAUE and one of its pharmacological mechanisms of DNA damage involves the inhibition of nuclear factor κB (NF-κB) (13). Caffeic acid derivatives block NF-κB activation (7), and it has been hypothesized that NF-κB inhibitory molecules are clinically beneficial as single therapeutic agents or in combination with classical chemotherapeutic agents for the treatment of hematological malignancies (14). Therefore, CAUE may inhibit NF-κB in leukemia cells and damage DNA to trigger the induction of apoptosis. NF-κB regulates hTERT expression by binding to a site 350-bp upstream of the translational initiation site (15). In addition, it has been reported that telomerase directly regulates NF-κB-dependent genes in cancer cells (16). Thus, there is a close correlation between NF-κB and telomerase activity. The results of the present study indicate that CAUE inhibits telomerase activation via mediation of hTERT protein expression, therefore, we hypothesize that the inhibition by CAUE is dependent on the inhibition of NF-κB activation.

In conclusion, CAUE inhibits DNA synthesis and suppresses telomerase activity. Targeting the inhibition of telomerase has been hypothesized to be beneficial for cancer chemotherapy due to its selectivity against malignant cells, thereby reducing side-effects. Telomerase inhibition is likely to be tested on humans in the future, in order to treat lymphoid cancers, including B-cell leukemia (17). The observations of the present study may therefore aid the development of therapeutic strategies for leukemia patients.
